# Enhancing Alzheimer Disease Detection Using Neuropsychiatric Symptoms: The Role of Mild Behavioural Impairment in the Revised NIA-AA Research Framework

**DOI:** 10.1177/08919887251366634

**Published:** 2025-08-13

**Authors:** Rebeca Leon, Maryam Ghahremani, Dylan X. Guan, Eric E. Smith, Henrik Zetterberg, Zahinoor Ismail

**Affiliations:** 1 Hotchkiss Brain Institute, 2129 University of Calgary, Calgary, AB, Canada; 2 Department of Psychiatry, 2129 University of Calgary, Calgary, AB, Canada; 3 Department of Clinical Neurosciences, 2129 University of Calgary, Calgary, AB, Canada; 4 Department of Community Health Sciences, 2129 University of Calgary, Calgary, AB, Canada; 5 Department of Psychiatry and Neurochemistry, Institute of Neuroscience and Physiology, The Sahlgrenska Academy at the University of Gothenburg, Mölndal, Sweden; 6 Clinical Neurochemistry Laboratory, Sahlgrenska University Hospital, Mölndal, Sweden; 7 Department of Neurodegenerative Disease, UCL Institute of Neurology, London, UK; 8 UK Dementia Research Institute at UCL, London, UK; 9 Hong Kong Center for Neurodegenerative Diseases, InnoHK, Hong Kong, China; 10 Wisconsin Alzheimer’s Disease Research Center, University of Wisconsin School of Medicine and Public Health, University of Wisconsin-Madison, Madison, WI, USA; 11 O’Brien Institute for Public Health, 2129 University of Calgary, Calgary, AB, Canada; 12 Department of Pathology and Laboratory Medicine, 2129 University of Calgary, Calgary, AB, Canada; 13 Clinical and Biomedical Sciences, Faculty of Health and Life Sciences, University of Exeter, Exeter, UK

**Keywords:** alzheimer disease, core 1 biomarkers, biomarker profiles, neuropsychiatric symptoms, mild behavioural impairment

## Abstract

**Background:**

As the prevalence of Alzheimer disease (AD) rises, early identification of at-risk individuals is essential for effective intervention. Mild behavioral impairment (MBI), which captures emergent and persistent neuropsychiatric symptoms (NPS) in later life, may enhance early detection of AD; however, its associations with 2024 NIA-AA Core 1 biomarkers remain unexplored. We investigated associations between MBI and cerebrospinal fluid (CSF) amyloid β-42 (Aβ42) and phosphorylated tau-181 (p-tau181).

**Method:**

Baseline data from 1327 dementia-free Alzheimer’s Disease Neuroimaging Initiative (ADNI) participants were analyzed. Participants were classified as MBI, non-MBI NPS, or no NPS. Gaussian mixture modeling defined biomarker positivity. Logistic and multinomial logistic regressions modeled associations between NPS status and biomarker positivity or biomarker profiles, adjusting for age, sex, education, and cognition.

**Results:**

MBI was associated with Aβ42+ (aOR = 2.26; 95% CI = 1.71-2.99), p-tau181+ (aOR = 1.72; 95% CI = 1.30-2.28), and AD continuum profile (aOR = 2.33; 95% CI = 1.73-3.14), but not with non-AD pathology. Non-MBI NPS showed no associations.

**Conclusion:**

MBI may serve as a behavioral marker of AD pathology.

## Introduction

In 2018, the National Institute on Aging–Alzheimer’s Association (NIA-AA) introduced a research framework to define Alzheimer disease (AD) biologically through a biomarker continuum and to operationalize clinical progression across several stages from cognitively unimpaired (CU) through dementia.^1^ This framework has since been updated, refining the criteria for diagnosing and staging AD.^2^ The 2024 revised guidelines introduce Core 1 biomarkers as the most important for early detection of AD neuropathologic changes (ADNPC). Core 1 biomarkers include amyloid positron emission tomography (Aβ-PET) and validated cerebrospinal fluid (CSF) and plasma biomarkers (amyloid beta-42 (Aβ42), phosphorylated tau-217 (p-tau217), p-tau181, p-tau231). These biomarkers become abnormal around the same time as Aβ-PET and can detect AD in both symptomatic and asymptomatic individuals.^2^ Notwithstanding some controversy regarding the diagnosis of AD in the absence of cognitive symptoms,^3^ these biomarkers are recognized in the revised guidelines as sufficient for AD diagnosis and clinical decision-making.

Despite advances in the biological definition of AD and the role of biomarkers, understanding the clinical manifestations is essential for appreciating AD across the clinical continuum. Cognitive decline in AD is well documented, progressing from CU to subjective cognitive decline (SCD) and mild cognitive impairment (MCI).^4,5^ Neuropsychiatric symptoms (NPS), nearly ubiquitous in AD dementia, often emerge early in the disease course and are also considered core symptoms.^6^ In approximately 30% of AD cases, NPS emerge before a formal cognitive diagnosis.^7^ However, under conventional nosology, differentiating NPS related to underlying AD from those due to psychiatric aetiologies, other medical conditions, or psychosocial factors is challenging.^8^

Thus, the Neuropsychiatric Syndromes Professional Interest Area of the International Society to Advance Alzheimer’s Research and Treatment (ISTAART) developed diagnostic criteria for mild behavioural impairment (MBI).^9^ The primary goal of these criteria was to better identify NPS associated with incident cognitive decline and dementia, potentially reflecting underlying neurodegeneration. To achieve this goal, MBI incorporates symptom natural history into assessment, which is absent in Diagnostic and Statistical Manual of Mental Disorders (DSM)-based nosologies. The MBI criteria stipulate that in older adults with normal cognition, SCD, or MCI, symptoms emerge *de novo* in later life, persist, and represent a change from longstanding behaviour or personality. Importantly, changes in cognition and behaviour, as per MBI, are not mutually exclusive; both cognitive status and MBI status must be considered to optimize risk stratification. Indeed, MBI proposes the same natural approach for behaviour as is currently used for cognition, i.e., new-onset persistent cognitive symptoms. Accordingly, MBI may serve as a potential marker of preclinical or prodromal AD in some individuals.^9^ An increasing number of studies have demonstrated the prognostic value of MBI in relation to dementia.^10-13^

A growing body of evidence has established a link between MBI and AD proteinopathies, highlighting the potential of MBI screening as a tool to improve early AD detection. Previous studies have shown both cross-sectional and longitudinal associations between MBI and CSF and plasma biomarkers, including Aβ42, p-tau181,^14^ p-tau 217, and total tau (t-tau).^15-19^ Importantly, individuals with NPS not meeting MBI criteria (non-MBI NPS) often show little to no significant difference in biomarker levels compared to those without NPS, underscoring the specificity of MBI for AD pathology over conventionally measured NPS.^15,19^ However, further research is needed, particularly in light of the 2024 revised AD criteria.^2^ Additionally, there is a need to demonstrate the clinical relevance of MBI, with dichotomization of +/− biomarker status to inform decision-making. The present study investigated the association between MBI and Core 1 AD biomarkers and profiles as defined in the 2024 NIA-AA criteria. We hypothesized that participants exhibiting MBI would be more likely to fall along the AD continuum compared to those without MBI, underscoring the value of MBI screening as an accessible marker for early AD detection.

## Methodology

### Study Participants

Participant data were obtained from the September 2024 data release of the Alzheimer’s Disease Neuroimaging Initiative (ADNI) (ADNI: adni.loni.usc.edu). Launched in 2003, ADNI is a collaborative effort involving multiple centers across North America that aims to track participants through periods of cognitive decline and dementia by evaluating biomarkers, neuroimaging, and neuropsychological status. ADNI’s inclusion criteria require participants to be 55-90 years old, have a Hachinski Ischemic Score of 4 or lower, and a Geriatric Depression Scale score of <6. Participants must also demonstrate adequate visual and auditory acuity for neuropsychological testing, maintain good general health without any conditions that would prevent enrollment, and have at least a sixth-grade education. For a complete list of inclusion and exclusion criteria by cognitive category, refer to the ADNI protocol (https://adni.loni.usc.edu/wp-content/themes/freshnews-dev-v2/documents/clinical/ADNI-1_Protocol.pdf).

ADNI Conversion Committee reviews participant reports and provides a consensus diagnosis. Details of diagnoses have been previously described elsewhere (https://adni.loni.usc.edu/wp-content/uploads/2010/09/ADNI_GeneralProceduresManual.pdf). All ADNI participants provided informed consent to participate, and the ethics committee approval to conduct this study was received at contributing ADNI sites. The investigators within ADNI (https://adni.loni.usc.edu/wp-content/uploads/how_to_apply/ADNI_Acknowledgement_List.pdf) contributed to the design and implementation of ADNI and/or provided data but did not participate in the analysis or writing of this report.

### Sample

Inclusion criteria were: (1) age ≥55 years; (2) available CSF Aβ42 and p-tau181 measures; (3) no dementia at baseline; (4) complete baseline Neuropsychiatric Inventory (NPI) or Neuropsychiatric Inventory Questionnaire (NPI-Q) data^20^; and (5) available demographic data required for modeling (Supplemental Figure 1). The final sample included participants with the following neurocognitive status: cognitively unimpaired (CU), defined by a Clinical Dementia Rating (CDR) global score and memory box score of 0; and MCI, defined by a CDR global score of 0.5 and memory box score of at least 0.5. The Mini-mental State Examination (MMSE) and Wechsler Logical Memory II sub-scale were also used to establish a CU or MCI diagnosis, with specific cut-off scores based on educational level. Further details can be found in the procedures manuals on the ADNI documentation page (https://adni.loni.usc.edu/help-faqs/adni-documentation/).

### MBI Operational definition

The presence and severity of NPS were evaluated using the NPI or NPI-Q,^20^ utilizing only the NPI domain severity score, excluding frequency, to ensure consistency across the 2 instruments. The NPI and its derivatives were originally designed to capture NPS in dementia populations over a one-moth reference period, shorter than the 6-month requirement of the MBI persistence criterion. Thus, we applied 2 validated algorithms to derive MBI symptom severity and persistence status.^21,22^ The severity algorithm utilized 10 of the 12 NPI/NPI-Q domains, mapped onto MBI domains. MBI decreased motivation domain was derived from the apathy/indifference item; emotional dysregulation from depression/dysphoria, anxiety, and elation/euphoria items; impulse dyscontrol from agitation/aggression, irritability/lability, and aberrant motor behaviour items; social inappropriateness from the disinhibition item; and psychosis from delusions and hallucinations items. The total MBI score was computed as the sum of scores across the 5 domains, resulting in a total score ranging 0-30. Higher scores indicated greater severity. The persistence algorithm determined whether the MBI symptom persistence criterion was met.^21^ Participants were classified as MBI if baseline total MBI score was ≥1 and persisted across at least two-thirds of all dementia-free study visits, which were 6-12 months apart. Those not meeting the symptom persistence criterion were classified as non-MBI NPS, while participants with a total score of 0 were classified as no NPS.

## Cerebrospinal Fluid Biomarkers

CSF samples were collected between September 7, 2005 and July 25, 2016. Roche Elecsys immunoassays were used to measure CSF Aβ42 and p-tau181 concentrations at the University of Pennsylvania (See the Methods document, “UPENN CSF Biomarkers Roche Elecsys Methods [ADNI1,GO,2,3]”, available to authorised users on the ADNI Image and Data Archive platform (https://ida.loni.usc.edu/login.jsp/). These biomarkers were classified into categories reflecting Aβ deposition and pathologic tau status, as per the 2024 NIA-AA revised criteria.^2^ Individuals without biomarker alterations were classified as having normal AD biomarkers (A- T_1_-). Those with evidence of Aβ deposition (low CSF Aβ42), with or without elevated tau (A+ T_1_- or A+ T_1_+), were assigned the AD continuum label. Individuals lacking evidence of Aβ deposition but with elevated CSF p-tau181 were classified as having non-AD pathologic change (A- T_1_+) (Supplemental Table 1).

## Statistical Analysis

All analyses were performed using RStudio (version 2024.09.0 + 375). Baseline sample characteristics were compared using two-sample t-tests for continuous variables and Chi-square test for categorical variables. Continuous variables with a normal distribution were presented as mean (standard deviation, SD). Biomarker levels, with skewed distributions or outliers, were summarized using median and interquartile range (IQR). Winsorization was applied to the biomarker measures to reduce the impact of outliers by replacing extreme values with the 5th or 95th percentile values.

**Figure 1.**
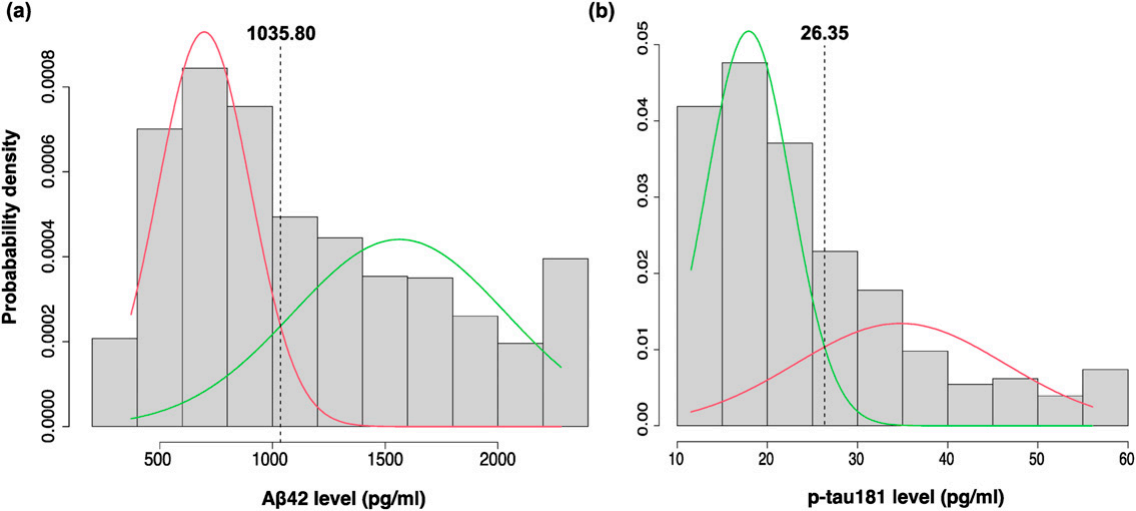
Gaussian distributions fitted using the Gaussian mixture model (GMM) to determine data-driven cut-offs for biomarker positivity for (A) Aβ42 and (B) p-tau181 CSF biomarkers at baseline. The vertical dashed lines represent intersection points corresponding to cut-off values of 1035.80 pg/mL for Aβ42 (A) and 26.35 pg/mL for p-tau181 (B).

Gaussian mixture modeling (GMM) was employed to establish a biomarker positivity cut-off point using the *mixtools* package. GMM is a statistical approach that assumes a dataset is generated from a mixture of several Gaussian distributions, each representing a distinct subgroup. By fitting multiple Gaussian distributions to the biomarker data, GMM can identify cut-off points where the biomarker characteristics change significantly.^23,24^ The optimal number of distributions required for data characterization in our sample was 2, determined using parametric bootstrapping. The biomarker positivity cut-off point was identified at the intersection of the 2 fitted normal distributions (Figure 1).

Logistic regressions, adjusted for age, sex, years of education, and MMSE score, modeled the associations between NPS status (exposure) and CSF biomarker positivity status (outcome), as determined by data-driven cut-off points. Subsequently, multinomial logistic regression, adjusted for the same covariates, was implemented to assess the relationship between NPS status (exposure) and AD biomarker profile, categorized as normal, AD continuum, and non-AD pathologic change (outcome). The goal of this secondary model was to determine whether MBI is more strongly associated with the amyloid or tau component of AD, or both, providing a more nuanced understanding of the relationship of MBI with distinct biomarker categories.

## Results

### Demographic Characteristics

The final sample comprised 1327 dementia-free participants (mean age 72.3 ± 7.1, 48.2% female). Cognitively, 348 (26.2%) were classified as CU and 979 (73.8%) as MCI. MBI status was determined using the NPI in 914 participants and the NPI-Q in 413 participants. Overall, 824 (62.1%) had no NPS, 160 (12.1%) had non-MBI NPS, and 343 (25.8%) met criteria for MBI. Compared to those without NPS, participants with MBI had a lower proportion of females (*P* < 0.001). MCI diagnosis was more prevalent in both the MBI and non-MBI NPS groups, compared to no NPS (*P* < 0.001). Furthermore, compared to no NPS, the non-MBI NPS group was younger (*P* = 0.009). Participants with MBI exhibited lower MMSE scores (*P* < 0.001), lower CSF Aβ42 levels (*P* < 0.001), and higher CSF p-tau181 levels (*P* < 0.001). In contrast, the non-MBI NPS group showed no significant differences in biomarker levels compared to no NPS. With Aβ42 and p-tau181 levels dichotomized based on data-driven cut-offs, participants with MBI had a higher percentage of A+ and T_1_+ cases (*P* < 0.001) and were more likely to exhibit an AD-continuum profile compared to no NPS (*P* < 0.001). In contrast, the biomarker profile of the non-MBI NPS group did not differ significantly from no NPS (Table 1).

**Table 1.** Sample Characteristics at Baseline. The No NPS Group Served as the Reference Group. P-values Were Calculated Using a Two-Sample t-test for Continuous Variables and Chi-Square Test for Categorical Variables. Bold P-values Indicate Statistical Significance (*P* < 0.05).

	No NPS(N = 824)	Non-MBI NPS (N = 160)	MBI (N = 343)	Non-MBI NPS *P*-value^1^	MBI *P*-value^1^
Age					
Mean (SD)	72.5 (6.9)	70.8 (7.7)	72.3 (7.1)	**0.009**	0.565
Sex					
Female	444 (53.9%)	81 (50.6%)	114 (33.2%)	0.503	**<0.001**
Male	380 (46.1%)	79 (49.4%)	229 (66.8%)		
Education					
Mean (SD)	16.3 (2.66)	16.4 (2.45)	16.2 (2.64)	0.622	0.405
MMSE					
Mean (SD)	28.6 (1.58)	28.3 (1.78)	27.9 (1.90)	0.063	**<0.001**
Clinical diagnosis					
CU	299 (36.3%)	31 (19.4%)	18 (5.2%)	**<0.001**	**<0.001**
MCI	525 (63.7%)	129 (80.6%)	325 (94.8%)		
Aβ42 level					
Median [IQR]	1132.0 [727.4, 1644.5]	1062.5 [704.2, 1573.3]	805.2 [586.1, 1204.0]	0.708	**<0.001**
Aβ42 status					
A-	446 (54.1%)	82 (51.3%)	109 (31.8%)	0.561	**<0.001**
A+	378 (45.9%)	78 (48.8%)	234 (68.2%)		
p-tau181 level					
Median [IQR]	20.7 [15.7, 28.1]	21.0 [15.2, 30.4]	23.7 [16.8, 33.7]	0.780	**<0.001**
p-tau181 status					
T_1_-	582 (70.6%)	105 (65.6%)	196 (57.1%)	0.243	**<0.001**
T_1_+	242 (29.4%)	55 (34.4%)	147 (42.9%)		
Profiles					
Normal	368 (44.7%)	65 (40.6%)	89 (25.9%)	0.631	**<0.001**
AD continuum	378 (45.9%)	78 (48.8%)	234 (68.2%)		
Non-AD pathology	78 (9.5%)	17 (10.6%)	20 (5.8%)		

Note: ^1^The reference group for the statistical tests was the no NPS group.

Abbreviations: AD = Alzheimer’s Disease; CU = Cognitively Unimpaired; IQR = Interquartile Range; MBI = Mild Behavioural Impairment; MCI = Mild Cognitive Impairment; NPS = Neuropsychiatric Symptoms; SD = Standard Deviation.

### Cross-Sectional Association of MBI with CSF Biomarkers and Biomarker Categories

Using parametric bootstrap with *k* = 1000 resamples, a bimodal distribution was identified with a cut-off point of 1035.80 pg/mL for Aβ42, and 26.35 pg/mL for p-tau-181 (Figure 1). Adjusted logistic regression models demonstrated that, compared to no NPS, MBI was significantly associated with Aβ42 positivity (adjusted OR [aOR] = 2.26, 95% CI = 1.71-2.99, *P* < 0.001) and p-tau181 positivity (aOR = 1.72, 95% CI = 1.30-2.28, *P* < 0.001) (Table 2), while non-MBI NPS did not show any significant association with either biomarker (Aβ42: aOR = 1.11, 95% CI = 0.78-1.58, *P* = 0.548; p-tau181: aOR = 1.31, 95% CI = 0.90-1.91, *P* = 0.156). Multinomial logistic regression revealed a significant association between MBI status and the AD continuum profile (aOR = 2.33, 95% CI = 1.73-3.14, *P* < 0.001), while no significant association was observed between MBI and the non-AD pathologic change profile (aOR = 1.16, 95% CI = 0.66-2.03, *P* = 0.611). Breaking down the AD continuum profile into its biomarker categories (A+ T_1_- and A+ T_1_+) revealed that while MBI status was significantly associated with both categories compared to A- T_1_- (i.e., normal), the association was considerably stronger with A+ T_1_+ (aOR = 2.95, 95%CI = 2.07-4.21, *P* < 0.001) than A+ T_1_- (aOR = 1.93, 95% CI = 1.37-2.71, *P* < 0.001). The non-MBI NPS group showed no significant association with the AD continuum profile (aOR = 1.20, 95% CI = 0.83-1.75, *P* = 0.333), its biomarker categories (A+ T_1_-: aOR = 1.08, 95% CI = 0.70-1.68, *P* = 0.722; A+ T_1_+: aOR = 1.32, 95% CI = 0.82-2.12, *P* = 0.248), or with non-AD pathologic change (aOR = 1.42, 95% CI = 0.78-2.59, *P* = 0.252) (Table 3).

**Table 2.** Cross-Sectional Associations Between NPS Status and CSF Biomarker Positivity (A+, T1+) Modeled Using Logistic Regression. Models Adjusted for Age, Sex, Years of Education, and Mini-Mental State Examination (MMSE) Score. The Reference Group for NPS Status was No NPS. Bold P-values Indicate Statistical Significance (*P* < 0.05).

Exposure	Odds ratios	95% CI	*P-value*	Outcome
NPS status [non-MBI NPS]	1.11	0.78–1.58	0.548	Aβ42 positivity (A+)
NPS status [MBI]	2.26	1.71–2.99	**<0.001**	Aβ42 positivity (A+)
NPS status [non-MBI NPS]	1.31	0.90–1.91	0.156	p-tau181 positivity (T_1_+)
NPS status [MBI]	1.72	1.30–2.28	**<0.001**	p-tau181 positivity (T_1_+)

Abbreviations: MBI = Mild Behavioural Impairment; NPS = Neuropsychiatric Symptoms; CI = Confidence Interval.

**Table 3.** Cross-Sectional Association Between NPS Status and Core 1 AD Biomarker Profiles, Normal, AD Continuum, and Non-AD Pathologic Change, Modeled Using Multinomial Logistic Regression. Models Adjusted for Age, Sex, Years of Education, and Mini-Mental State Examination (MMSE) Score. The Reference Group for NPS Status was No NPS. Bold P-values Indicate Statistical Significance (*P* < 0.05).

Exposure	Odds Ratios	95% CI	*P-value*	Outcome
NPS status [non-MBI NPS]	1.20	0.83–1.75	0.333	AD continuum
	1.08	0.70 – 1.68	0.722	A+ T_1_-
	1.32	0.82 – 2.12	0.248	A + T_1+_
NPS status [MBI]	2.33	1.73–3.14	**<0.001**	AD continuum
	1.93	1.37 – 2.71	**<0.001**	A+ T_1_-
	2.95	2.07 – 4.21	**<0.001**	A+ T_1+_
NPS status [non-MBI NPS]	1.42	0.78–2.59	0.252	Non-AD pathologic change
NPS status [MBI]	1.16	0.66–2.03	0.611	Non-AD pathologic change

Abbreviations: MBI = Mild Behavioural Impairment; NPS = Neuropsychiatric Symptoms; CI = Confidence Interval, AD = Alzheimer’s Disease.

## Discussion

In a sample of 1327 dementia-free participants from ADNI, we found a significant association between MBI and NIA-AA CSF Core 1 AD biomarkers. Participants exhibiting MBI had higher odds of Aβ42 (A+) and p-tau181 (T_1_+) positivity based on data-driven cut-offs. Furthermore, MBI was associated with biomarker profiles consistent with the AD continuum (A+ T_1_+ or A+ T_1_-), while no association was observed between MBI and the non-AD pathological change profile (A- T_1_+). These findings suggest that MBI serves as a critical behavioural marker for identifying individuals along the AD continuum, a distinction not observed in conventionally measured NPS that do not meet MBI criteria (non-MBI NPS).

Consistent with our findings, previous research using neuroimaging and biofluid biomarkers has highlighted the strong association between MBI-consistent NPS and AD biomarkers. In MCI participants from ADNI and MEMENTO, MBI was associated with lower baseline CSF Aβ42, higher p-tau181, and higher p-tau18/Aβ42 and t-tau/Aβ42 ratios, compared to no NPS.^19^ Longitudinally, MBI predicted higher levels of CSF p-tau181, and p-tau181/Aβ42 and t-tau/Aβ42 ratios over a 4-year period.^19^ These findings were consistent in both ADNI and MEMENTO cohorts. The non-MBI NPS group showed weaker and less consistent associations, with significant findings limited to the Aβ42/Aβ40 ratio cross-sectionally and t-tau longitudinally, both with considerably smaller effect sizes compared to MBI associations.^19^ The present study extends these findings to an earlier point along the cognitive continuum by including CU participants, employing a more robust approach to operationalize MBI, and integrating the latest biomarker classification and profiling framework. Importantly, by using a data-driven approach to dichotomize +/− biomarker status, findings are valid, clinically relevant, and interpretable, potentially informing clinical decision-making.

Complementary findings have been reported for the association between MBI and plasma biomarkers of AD. In dementia-free ADNI participants, MBI was associated with higher plasma p-tau181 levels, declining memory and executive function, and 3.92-fold greater 4-year hazard for dementia vs no NPS.^15^ The non-MBI NPS group showed no significant associations with plasma p-tau181.^15^ Similarly, in a recent study of older adults with SCD or MCI, those with MBI psychosis showed a steeper increase in plasma p-tau181.^18^ Additionally, higher MBI total scores were linked to a lower plasma Aβ42/Aβ40 ratio, with domain-specific associations found for MBI-affective dysregulation.^16^Most recently, an ADNI study using plasma p-tau217, the most accurate plasma biomarker reflecting both Aβ and tau pathology,^25^ reported that dementia-free older adults with MBI had significantly higher p-tau217 levels and greater odds of p-tau217 positivity compared to their MBI-counterparts.^14^

Several neuroimaging studies have added to the evidence base linking MBI to AD-related neuropathologic changes. Aβ-PET studies have reported associations between MBI and greater Aβ burden in CU^26^ and mixed CU/MCI participants.^27^ Tau-PET studies have shown that, among Aβ-positive individuals with normal cognition or MCI,^17,28^ MBI was associated with tau uptake in brain regions implicated early in AD. Magnetic resonance imaging (MRI) studies in dementia-free older adults have identified associations between MBI and neurodegeneration in early-stage AD regions.^29,30^ Neuropathological studies further validate MBI as a significant predictor of progression to neuropathologically-confirmed AD.^31^ These findings highlight the link between MBI and core AD pathologies, suggesting that MBI improves NPS assessment for identifying symptoms more likely to represent the behavioural manifestations of neurodegenerative disease.

In contrast, some studies found no association between MBI and AD biomarkers, although with methodological differences. In CU participants from the TRIAD cohort, MBI Checklist (MBI-C^32^) total scores were associated with greater Aβ-PET tracer uptake, but not tau-PET uptake.^26^ However, the cross-sectional nature of this study and its small sample size (n = 96), with few participants reporting behavioural symptoms (60.4% scored 0 on the MBI-C), may have contributed to the lack of significant findings for tau. Further, amyloid-tau interactions were not included in the modeling, which have since been shown to be an important factor in the association between MBI and tau.^28^ In dementia-free older adults from ADNI, both cross-sectionally and longitudinally, MBI was associated with higher Aβ tracer uptake but not tau burden (measured by tau-PET).^27^ However, this study used a single-visit approach to define MBI, failing to account for symptom persistence—a cardinal MBI criterion, linked to a higher likelihood of progression from MCI to dementia and a lower likelihood of reversion to NC.^10^ Our study adopted a natural history approach to identify relevant NPS, rather than relying on cross-sectional symptom assessment. Notably, this natural history approach^21^ has been successfully applied in a recent study, demonstrating strong associations between MBI and incident dementia in CU older adults, including those with SCD.^12^

Our data-driven cut-offs were 1035.80 pg/mL for Aβ42 and 26.35 pg/mL for p-tau181 CSF biomarkers using the Roche Elecsys immunoassay. These cut-offs closely align with the Roche Elecsys published cut-offs of ≤1030 pg/mL for Aβ42 positivity and >27 pg/mL for p-tau181 positivity. In fact, repeating the analyses using the published cut-offs yielded very similar results (see Supplemental Tables 2 and 3), indicating that our approach and findings are robust. Previous studies using the same assays have reported cut-off values for Aβ42 ranging from 880 to 1100 pg/mL^33-38^ and for p-tau181 from 19.2 to 27 pg/mL^33,34,37-39^ pg/ml. However, in most of these studies, except for one involving community-dwelling volunteers,^38^ cut-off values were derived from samples that included participants across the cognitive continuum from CU to dementia, whereas our study focused solely on a dementia-free sample. Therefore, the exact cut-off points could vary depending on the study population. Furthermore, cut-off variability may also stem from differences in pre-analytic conditions, which have been shown to affect CSF biomarker measurements.^40^ Nonetheless, our cut-offs certainly fall within the range of values previously reported, providing reassurance.

Our study outlines important implications for both clinical care and research in AD, establishing MBI as a key behavioural marker for identifying individuals along the AD continuum—a distinction not consistently observed with conventional NPS measurement. The specificity of MBI to AD-related pathology, as opposed to non-AD pathologic changes, highlights its potential as a valuable tool for differentiating AD from other neurodegenerative conditions with similar behavioural symptoms. Furthermore, across biomarker profiles within the AD continuum, MBI was significantly linked to both A+ T_1_- and A+ T_1_+, with a stronger association observed with A+ T_1_+. Thus, even in the absence of tau pathology, the presence of MBI suggests that the AD may be present. These findings could have implications for future clinical trials, where MBI assessments could serve as an accessible, scalable, and even remotely administered screening approach to identify those at higher risk of Core 1 AD biomarker positivity. This sample enrichment would improve screening efficiency for eligible clinical trial participants. Clinically, assessing for MBI could enable earlier intervention, with lifestyle modification and dementia prevention and risk reduction approaches, and possible consideration of AD disease modifying therapies, if appropriate.

We acknowledge study limitations including the use of NPI/NPI-Q data to extrapolate MBI. The two-thirds-visits approach^21^ operationalized the MBI symptom persistence criterion, but may not have captured behavioural changes persisting beyond the 4-week reference range of the NPI/NPI-Q. The use of the MBI-C to capture MBI can mitigate this limitation. The MBI-C is the gold standard for measuring MBI, designed to capture a broader spectrum of mild NPS typically observed in pre-dementia stages over a 6-month reference period.^32,41^ Another key limitation of our study is the use of Aβ42 alone to represent the A biomarker category, rather than the Aβ42/Aβ40 ratio, which has been shown to improve diagnostic accuracy for AD.^42^ However, in our dataset, CSF Aβ40 measurements were unavailable for nearly 70% of participants, limiting the feasibility of using the Aβ42/Aβ40 ratio. Future studies with more complete CSF biomarker data, including Aβ40, are needed to assess the potential benefits of using the Aβ42/Aβ40 ratio. Additionally, employing the ratio may provide a more accurate characterization of the A-T+ group, as its prevalence is reportedly lower when using Aβ42/Aβ40 instead of Aβ42.^43^ Another limitation is the ADNI inclusion criteria, which select relatively healthy older adults with intact sensory function and few comorbidities, potentially limiting generalizability to typical geriatric patients. Nonetheless, existing evidence supports robust associations between MBI and AD biomarkers across more clinically diverse populations.^19,44^ Future studies should confirm our findings in broader samples. While our study offers an initial cross-sectional exploration of the relationship between Core 1 AD biomarkers and MBI, longitudinal research is required to compare biomarker trajectories in individuals with and without MBI. Additionally, domain-specific analyses of MBI are warranted, as accumulating evidence indicates that individual domains confer varying levels of risk for dementia. For instance, MBI apathy,^45^ affective dysregulation,^46^ and psychosis^47,48^ have each been independently linked to incident dementia, with psychosis demonstrating the highest hazard ratio. A recent study found that MBI apathy was associated with CSF p-tau181, p-tau181/Aβ42, and t-tau181/Aβ42 both cross-sectionally and longitudinally.^49^ Further exploration of MBI domain-level biomarker associations is critical to delineate the relative contributions of each domain to the overall MBI construct. However, such analyses will require substantially larger samples to ensure sufficient statistical power.

## Conclusion

MBI can serve as a behavioural marker of AD pathology, enhancing our understanding of how NPS are related to biomarkers in the disease continuum. Incorporating MBI assessment into clinical workflows could improve early detection, facilitate more accurate diagnosis, and enable more personalized and targeted clinical management for individuals at risk. Furthermore, the methodological advancements in the study could enhance the design of future research, ultimately contributing to the development of new biomarkers and treatments for AD.

## Supplemental Material


Supplemental Material - Enhancing Alzheimer Disease Detection Using Neuropsychiatric Symptoms: The Role of Mild Behavioural Impairment in the Revised NIA-AA Research Framework
Supplemental Material for Enhancing Alzheimer Disease Detection Using Neuropsychiatric Symptoms: The Role of Mild Behavioural Impairment in the Revised NIA-AA Research Framework by Rebeca Leon, Maryam Ghahremani, Dylan X. Guan, Eric E. Smith, Henrik Zetterberg, Zahinoor Ismail in Journal of Geriatric Psychiatry and Neurology

## Data Availability

Data used in preparing this manuscript are publicly available upon request from ADNI (https://adni.loni.usc.edu/data-samples/adni-data/).
