# Supplementary material for: Enhancing Alzheimer Disease Detection Using Neuropsychiatric Symptoms: The Role of Mild Behavioural Impairment in the Revised NIA-AA Research Framework
Source: J Geriatr Psychiatry Neurol. 2025 Aug 13;39(3):277–87. doi: 10.1177/08919887251366634 (PMC13009234; doi:10.1177/08919887251366634)

Supplementary materials

Supplementary Table 1. Core 1 AD biomarkers and their categorization. Abbreviations: AD= Alzheimer’s disease; CSF: cerebrospinal fluid.

<table>
<tr><th>Core 1 AD biomarkers</th><th colspan="2">CSF analytes used in the analysis</th></tr>
<tr><td>A (Aβ proteinopathy)
T_1: (phosphorylated and secreted AD tau)</td><td colspan="2">Aβ42
p-tau181</td></tr>
<tr><th>Biomarker profiles</th><th colspan="2">Biomarker categories</th></tr>
<tr><td>A-T_1-</td><td colspan="2">Normal AD biomarkers</td></tr>
<tr><td>A+ T_1-</td><td>Alzheimer’s pathologic change</td><td rowspan="2">Alzheimer’s continuum</td></tr>
<tr><td>A+ T_1+</td><td>Alzheimer’s disease</td></tr>
<tr><td>A- T_1+</td><td colspan="2">Non-AD pathologic change</td></tr>
</table>

Supplementary Table 2. Cross-sectional associations between NPS status and CSF biomarker positivity (A+, T1+) based on the published Roche Elecsys cut-off values (≤1,030 pg/mL for Aβ42 positivity and >27 pg/mL for p-tau181 positivity). Models adjusted for age, sex, years of education, and Mini-Mental State Examination (MMSE) score. The reference group for NPS status was No NPS.

Exposure	*Odds Ratios*	*95% CI*	*p*	*Outcome*
NPS status [non-MBI NPS]	1.12	0.79 – 1.59	0.531	Aβ42 positivity (A+)
NPS status [MBI]	2.24	1.70 – 2.97	**<0.001**	Aβ42 positivity (A+)
NPS status [non-MBI NPS]	1.28	0.87 – 1.88	0.201	p-tau181 positivity (T_1+)
NPS status [MBI]	1.85	1.40 – 2.46	**<0.001**	p-tau181 positivity (T_1+)

Supplementary Table 3. Cross-sectional association between NPS status and Core 1 AD biomarker profiles, normal, AD continuum, and non-AD pathologic change, defined based on the published Roche Elecsys cut-off values (≤1,030 pg/mL for Aβ42 positivity and >27 pg/mL for p-tau181 positivity). Models adjusted for age, sex, years of education, and Mini-Mental State Examination (MMSE) score.

Exposure	*Odds Ratios*	*95% CI*	*p*	*Outcome*
NPS status [non-MBI NPS]	1.19	0.82 – 1.73	0.354	AD continuum
	1.09	0.71 – 1.67	0.692	A+ T_1-
	1.31	0.81 – 2.11	0.266	A+ T_{1+}
NPS status [MBI]	2.35	1.74 – 3.16	**<0.001**	AD continuum
	1.90	1.36 – 2.66	**<0.001**	A+ T_1-
	3.11	2.18 – 4.45	**<0.001**	A+ T_{1+}
NPS status [non-MBI NPS]	1.37	0.73 – 2.56	0.330	Non-AD pathologic change
NPS status [MBI]	1.28	0.73 – 2.26	0.392	Non-AD pathologic change

Supplementary Figure 1. Flowchart illustrating inclusion/exclusion criteria for selecting the study sample from the ADNI dataset. Abbreviations: CSF= cerebrospinal fluid; NPI= Neuropsychiatric Inventory; NPI-Q= Neuropsychiatric Inventory Questionnaire.

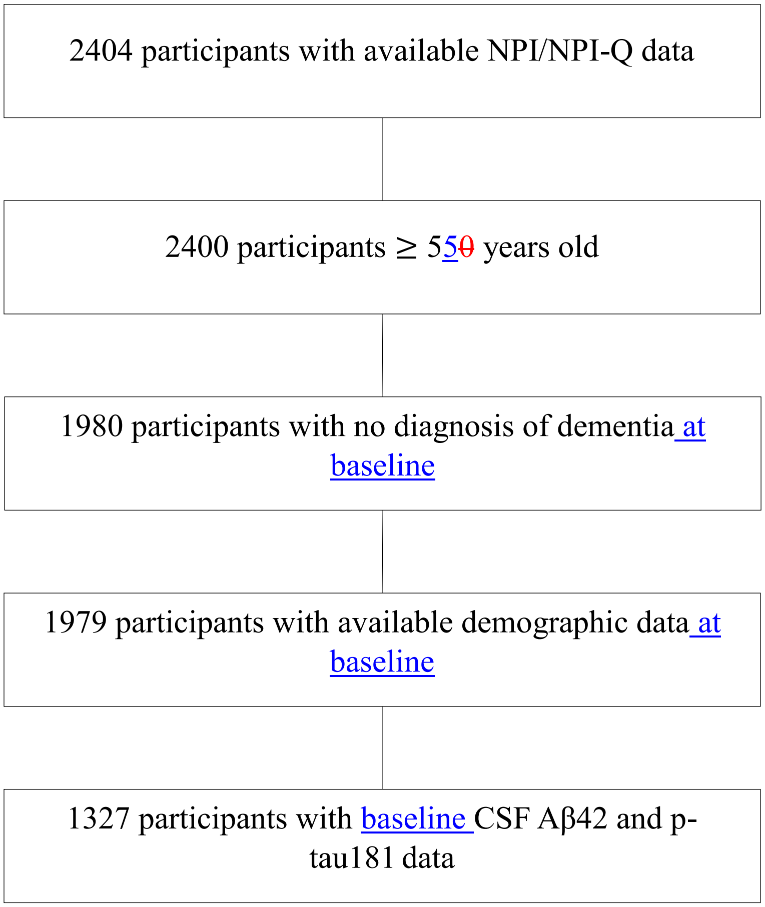

Supplement: Supplemental Material - Enhancing Alzheimer Disease Detection Using Neuropsychiatric Symptoms: The Role of Mild Behavioural Impairment in the Revised NIA-AA Research Framework [file sj-pdf-1-jgp-10.1177_08919887251366634.pdf]
